# A structured model and likelihood approach to estimate yeast prion propagon replication rates and their asymmetric transmission

**DOI:** 10.1371/journal.pcbi.1010107

**Published:** 2022-07-01

**Authors:** Fabian Santiago, Suzanne Sindi

**Affiliations:** 1 Department of Mathematics, University of Arizona, Tucson, Arizona, United States of America; 2 Department of Applied Mathematics, University of California Merced, Merced, California, United States of America; Pázmány Péter Catholic University: Pazmany Peter Katolikus Egyetem, HUNGARY

## Abstract

Prion proteins cause a variety of fatal neurodegenerative diseases in mammals but are generally harmless to Baker’s yeast (*Saccharomyces cerevisiae*). This makes yeast an ideal model organism for investigating the protein dynamics associated with these diseases. The rate of disease onset is related to both the replication and transmission kinetics of propagons, the transmissible agents of prion diseases. Determining the kinetic parameters of propagon replication in yeast is complicated because the number of propagons in an individual cell depends on the intracellular replication dynamics and the asymmetric division of yeast cells within a growing yeast cell colony. We present a structured population model describing the distribution and replication of prion propagons in an actively dividing population of yeast cells. We then develop a likelihood approach for estimating the propagon replication rate and their transmission bias during cell division. We first demonstrate our ability to correctly recover known kinetic parameters from simulated data, then we apply our likelihood approach to estimate the kinetic parameters for six yeast prion variants using propagon recovery data. We find that, under our modeling framework, all variants are best described by a model with an asymmetric transmission bias. This demonstrates the strength of our framework over previous formulations assuming equal partitioning of intracellular constituents during cell division.

## Author summary

In this work we investigate the transmissible [*PSI*^+^] phenotype in yeast. The agents responsible for this phenotype are propagons, misfolded protein aggregates of a naturally occurring protein. These propagons increase in number within a cell and are distributed between cells during division. We use mathematical modeling to infer the replication rate of propagons within cells and if propagons are transmitted equally or unequally during cell division. Prior models in this area assumed only symmetric transmission when fitting replication rates. We couple this model with a novel likelihood framework allowing us to exclude influential outliers from our datasets when inferring parameters. We find that for all six protein variants we study, propagons are transmitted asymmetrically with different biases. Our results can be reproduced with the code and data available at https://github.com/FS-CodeBase/propagon_replication_and_transmission/.

## Introduction

Today in the United States, millions of individuals suffer from Alzheimer’s disease and other similar dementias. The cause of these diseases is thought to be the accumulation of misfolded proteins in the brain [1]. Currently, Alzheimer’s is the sixth-leading cause of death in the United States and the fifth-leading cause of death for individuals 65 and over [1]. Beyond Alzheimer’s, there are many other disorders caused by protein misfolding. Neurodegenerative diseases such as Parkinson’s and Huntington’s disease to less well-known diseases like Kuru and Creutzfeldt-Jakob Disease [2, 3], have different pathology but are characterized by the accumulation of amyloid aggregates. In addition, protein misfolding disorders have also been observed in other mammals such as scrapie in sheep, chronic wasting disease in deer and Bovine Spongiform Encephalopathy in cattle [4, 5]. Collectively, these diseases are untreatable and invariably fatal. While the details differ, all share two key commonalities: (1) a misfolded form of a protein *appears*; (2) the misfolded form of the protein forms aggregates, which then spread their folded confirmation through contact with other normally folded proteins. Because of the growing body of literature suggesting potential regulatory effects of prions (see [6] for example), in our work below we will refer to prion protein as alternatively folded protein instead of misfolded protein.

A promising biological system allowing for insight into alternatively folded mammalian protein and prion disease is the yeast *Saccharomyces cerevisiae*. A number of harmless, heritable phenotypes in yeast are shown to be transmitted vertically to new daughter cells by heritable prion elements termed propagons [4, 7]. Because these propagons are harmless to the yeast cells, researchers are able to study the protein dynamics themselves in the absence of harming the host. Indeed, biologists have many experimental tools that have been developed for yeast which allow for a detailed interrogation of the protein aggregation system in ways that are not possible to do in mammalian systems *in vivo* [6]. However, a unique challenge to working with yeast as a model system is that during the experimental time course, the yeast cells themselves continue to divide [8]. Without cell division, we know that the number of propagons in a cell will increase until it reaches a steady-state concentration where the propagon number is in balance with the soluble protein level [8–11]. However, the number of propagons in a cell will decrease when the cell divides as any propagons (i.e., transmissible aggregates, see [7] for more details) will be separated between the resulting mother and daughter cells [4, 8]. This means that for meaningful quantitative comparisons between mathematical models and experimental systems, care must be taken to understand the population averages but also heterogeneity amongst cells in the same population.

In this work, we consider not only the protein dynamics within yeast cells, but the cellular populations themselves. More specifically, in this work we develop a structured population model of yeast cells where the transmission of prion propagons are tracked as cells divide and as propagons replicate. We use our model to infer both the amplification rate and transmission bias of propagons from six prion variants (some are variants of [*PSI*^+^] and some are mutants, see [12] for full details). This work is not the first to use the structured population framework, nor to infer a quantitative rate for transmission bias. A structured population model was previously applied to propagon data in [13]. The transmission bias of propagons has been directly observed using propagon counting assays (see [14] for example). In 2009, Byrne et al. [15] used propagon curing data to quantitatively link the propagon transmission bias to the relative volumes of mother and daughter cells at cytokenesis. In comparison to these prior studies, our work offers several novel contributions. First, the model we develop here is more general, allowing for propagon amplification and asymmetric transmission. Second, our inference framework is more general. In contrast to [13], rather than assuming the variance in the propagon counts is proportional to the system mean, we employ the full likelihood of the data. In contrast to [15], we consider both propagon replication and transmission. Finally, we study a larger set of prion variants.

With this more general framework, we find that all six variants are best described by an asymmetric transmission of propagons between actively dividing yeast cells. With our model selection framework we were also able to exclude influential outliers from our prion variant datasets for computing kinetic parameters of prion variants. Moreover, we find differences among the variants for both the propagon replication rate and the transmission bias. Encouragingly, prion variants with similar phenotypic properties are fit with similar kinetic parameters. As such, our framework offers the ability to infer meaningful properties about prion variants even with our simplified model of intracellular propagon dynamics.

In the biological background section we develop the background of prion variants and the recovery assays we model. The methods section describes our propagon and generation structured population model and the likelihood approach we use for fitting kinetic parameters to experimental data. In the results section we first characterize the ability of our model and inference framework to recover the correct kinetic parameters and then apply our model and inference framework to recovery data from six distinct prion variants. In the discussion and conclusion section we discuss the implications of our study as well as factors to be considered in future studies on prion propagon dynamics.

### Biological background

As mentioned in the introduction, yeast prions were not discovered in the context of a disease but in one of mysterious heritable phenotypes [16, 17]. In addition, there is a considerably shorter history of knowledge about yeast prions than their mammalian counterparts [6, 9]. The [*PSI*^+^] phenotype in yeast that we now know to be linked to a prion form of the protein Sup35, was discovered in 1965 by biologist Brian Cox. The phenotype corresponded to that of a white colored colony and the ability to grow a colony on media lacking adenine [16]. Remarkably, this phenotype appeared to be heritable, but the nature of transmission was by some unknown nonchromosomal cytoplasmic element [18]. For years researchers searched for a DNA or RNA basis for [*PSI*^+^]. In 1994, Wickner, following insights from mammalian diseases [19, 20], hypothesized that [*PSI*^+^] and [*URE3*] (another phenotype whose determinant was mysterious) were propagated by an alternatively folded (prion) form of their respective proteins [21]. In 1996, Paushkin et al. [22] and Patino et al. [23], separately demonstrated that the [*PSI*^+^] phenotype was the result of an alternatively folded form of the protein Sup35 that was self-propagating. Today we know that many proteins in yeast are capable of forming prions and that a given prion protein may have multiple variants—distinct alternatively folded confirmations—each of which is capable of propagating through this self-propagation process. Indeed, it is possible that prions may, at least in yeast, offer beneficial functions including serving as heritable bet-hedging devices diversifying microbial phenotypes [24]. In this study, as mentioned in the introduction, we consider propagon amplification data from six variants of the [*PSI*^+^] prion in yeast [12].

For mammalian prion disease, the disease phenotype is observed at the level of a single organism (i.e., a cow, human, mouse, etc). However, in yeast prion biology the prion phenotypes are visible at the single-cell level with a different assay where the phenotype is observed with a yeast colony consisting of many cells founded by a single cell with the prion phenotype [25]. As such, it has been challenging to establish a precise link between the infective species, or propagon (that necessarily) reside in a single cell with the colony level phenotype [4]. While it is clear that the presence of a single propagon in the founding cell is necessary for the appearance of the prion phenotype at the colony level, it is not clear that it is sufficient [7]. In this work, following others [8, 13, 26] we will assume that the presence of a single propagon in a founding cell is both necessary and sufficient for the appearance of the prion phenotype at the colony level. As such, yeast prion dynamics are inherently a multi-scale process [27, 28].

**Fig 1:**
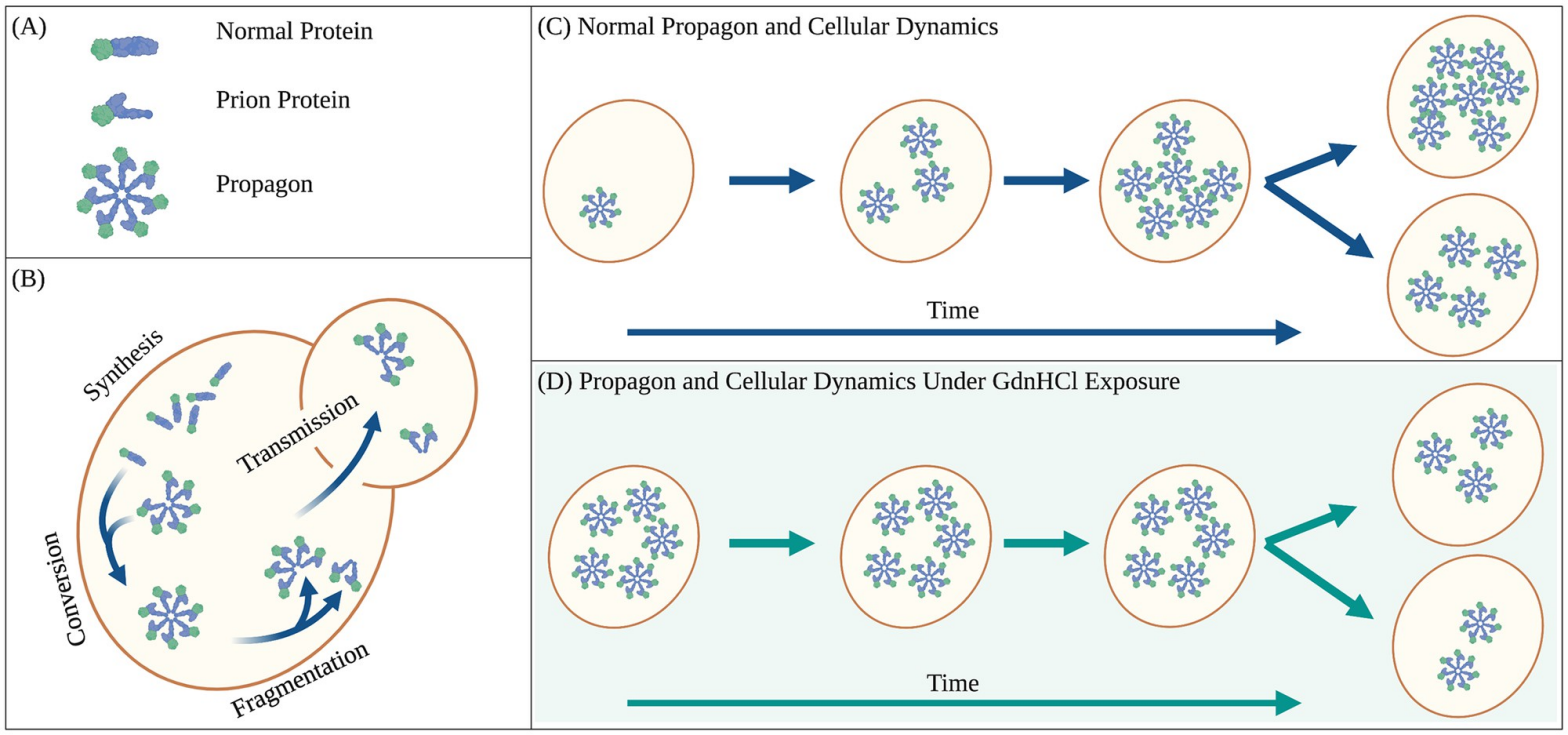
Multiscale yeast prion aggregate (propagon) dynamics. (A) Within each cell in the colony is a mixture of normal protein and prion (alternatively folded) protein. Prion proteins are contained in propagons of multiple alternatively folded monomers. (B) Within each cell normal protein is produced (synthesis) and incorporated into existing propagons which leads to conversion of the normal protein to the prion form and increases the size of a propagon. Aggregates may increase in number by fragmentation and must be spread from mother to daughter cells during division (transmission). (C) Under normal growth conditions, the number of propagons increases during the lifetime of a cell and is split during cell division. (D) When cells are grown under GdnHCl fragmentation is assumed to stop and the number of propagons remains unchanged during the lifetime of a cell.

Prion phenotypes occur when an alternatively folded form of a protein occurs, and rather than be cleared by cellular quality control machinery, the form persists and associates in aggregates (propagons), ordered structures of prion monomers as shown in Fig 1A. More specifically, four steps are essential to the maintenance of prion phenotypes (see Fig 1B). First, normal protein is continually produced by the cell. Second, prion propagons convert the normally folded protein to its alternatively folded confirmation through a templated conversion process when an aggregate incorporates the normally folded protein. This conversion process increases the size of the prion propagon. Third, the total number of propagons increases when the propagons are fragmented. This increases the total number of templating units and thus accelerates the conversion process. Fourth, propagons are transmitted between cells during division. Intriguingly, the cell division process creates an interesting phenomenon from the perspective of a single cell. Between cell divisions, the number of propagons increases, and it will then decrease when cell division occurs Fig 1C. Due to the low frequency of spontaneous [*PSI*^+^] appearance, ∼ 10^−8^ − 10^−7^/generation [29], in this work we assume there is no spontaneous appearance of propagons.

Yeast biologists take advantage of the fact that when cells are exposed to Guanadine Hydrochloride (GdnHCl) cell division is not impacted, but the propagon fragmentation process is assumed to halt. As such, the number of propagons within a cell, and indeed in the entire population is kept constant, while the number of cells in the colony continues to increase, see Fig 1D. This allows for experiments which probe the number of propagons in a single cell by regrowing colonies and assuming that any cell with at least one propagon will create a colony with a prion phenotype. A single yeast cell with propagons is introduced to a GdnHCl environment and the population of cells is allowed to grow normally. Because propagons will be split between mother and daughter cells during division, the expected number of propagons per cell will continue to decrease until the point where a cell in the population is extremely unlikely to have more than one propagon [26, 30, 31]. Then each cell in this population is allowed to form their own colony under normal growth conditions.

**Fig 2:**
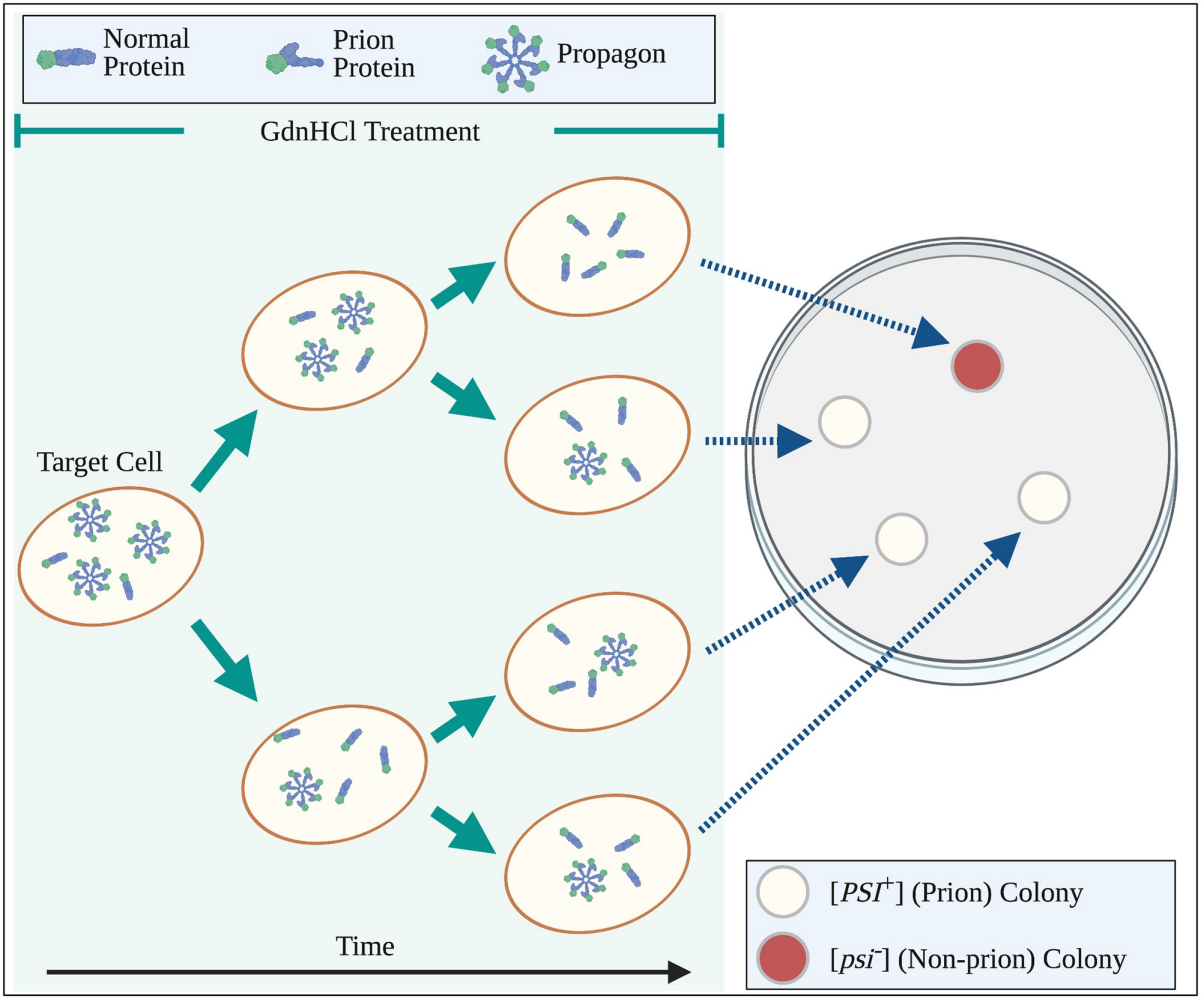
Propagon amplification assay. A two-step process is used to count the number of transmissible prion aggregates (propagons) in a single cell. *Left*: A single target cell is isolated, and propagon fragmentation is stopped through exposure to GdnHCl. Since propagons (pinwheels) cannot increase in number, they are diluted through cell division (green arrows). *Right*: After sufficient dilution, i.e. each yeast cell is likely to contain at most one propagon, the colony is replated onto solid media. In the absence of GdnHCl, each single cell serves as a founder of a distinct yeast colony. The number of propagons in the target cell corresponds to the number of white colonies in the plate.

In propagon recovery experiments, yeast biologists use GdnHCl exposure in two phases to observe the amplification of propagons (see Fig 2). In the first phase, yeast cells are treated with GdnHCl until the propagons have sufficiently diluted. In this first phase, as propagons present at the beginning of GdnHCl exposure will exist for all time, the expected number of propagons per cell will continue to decrease as cell division continues as normal. In time, cells in the colony will contain a very low number of propagons per cell, ideally one. In the second phase, the resulting cells from phase one are transferred to a GdnHCl free environment and are allowed to form individual colonies. The number of white colonies is then assumed to correspond exactly to the number of cells with at least one propagon (see Fig 2). This is because the resulting colony whose founding cell had at least one propagon will have the [*PSI*^+^] prion phenotype (white) while those founded by a cell with no propagons will have the [*psi*^−^] non-prion phenotype (red).

## Results

In this section we demonstrate that we can recover known parameters from simulated data using the adaptive Metropolis (AM) algorithm with our likelihood formulation, and study the effects of hourly sampling rate on these estimates. We then apply these parameter estimation methods to experimental data for six prion variants and perform model selection between symmetric division against asymmetric transmission of propagons during cell division.

### Parameter inference on simulated data

We first verify the capability of our likelihood formulation, Eq (16), and the AM algorithm 1, detailed in the methods to estimate known kinetic parameter values from simulated data before applying our methods to the experimental data. The simulated datasets are created using rejection sampling methods [32] on the asymmetric transmission of propagons (ATP) model presented in the methods. We take the cell division rates *α*_*i*_(*t*), to be constant during each period of cell division, with a rate of *α*_*i*_(*t*) = 0.46 hr^−1^, and that death is negligible throughout the duration of the experiment by setting *β*_*i*_(*t*) = 0. Furthermore, we assume the initial distribution of propagons to be a truncated normal distribution defined on the interval *R* = (0, ∞) with *μ* = 10 and *σ* = 1, that is $ϒ\left(a\right)=N^{+}\left(a;\mu =10,\sigma =1\right)$, to simulate a colony with low number of propagons at the beginning of the experiment.

**Algorithm 1** Adaptive Metropolis Algorithm

1: *D* ← load data

2: *L* ← load likelihood formulation

3: *M* ← load maximum number of iterations

4: *k* ← load non- adaptive period length

5: ***θ***^(0)^ ← initial value(s) for ***θ***

6: ***V***^(0:*k*)^ ← initial covariance matrix values for non-adaptive period *V*_0_

7: sp ← set covariance scaling design parameter *s*_*p*_

8: ep ← set identity scaling *ε* for positive definite *V*

9: **for** *i* = 1 **to** *k* **do**            ▹ Non-adaptive period

10:  ***θ***_*new*_ ← *N*(***θ***^(*i*−1)^, ***V***^(*i*−1)^)

11:  *u* ∼ *U*(0, 1)

12:  *α* ← min {1, *L*(***θ***_*new*_|*D*)/*L*(***θ***^(*i*−1)^|*D*)}

13:  **if** *u* < *α* **then**

14:   ***θ***^(*i*)^ ← ***θ***_*new*_

15:  **else**

16:   ***θ***^(*i*)^ ← ***θ***^(*i*−1)^

17: **for** *i* = *k* + 1 **to** *M* **do**            ▹ Adaptive period

18:  ***θ***_*new*_ ← *N*(***θ***^(*i*−1)^), ***V***^(*i*−1)^)

19:  *u* ∼ *U*(0, 1)

20:  *α* ← min {1, *L*(***θ***_*new*_|*D*)/*L*(***θ***^(*i*−1)^|*D*)}

21:  **if** *u* < *α* **then**

22:   ***θ***^(*i*)^ ← ***θ***_*new*_

23:  **else**

24:   ***θ***^(*i*)^ ← ***θ***^(*i*−1)^

25:  ***V***^(*i*)^ = sp * cov (***θ***^(0)^, ***θ***^(1)^, …, ***θ***^(*i*)^) + ep * *I*_*p*_

26: **return** ***θ***, *V*

**Fig 3:**
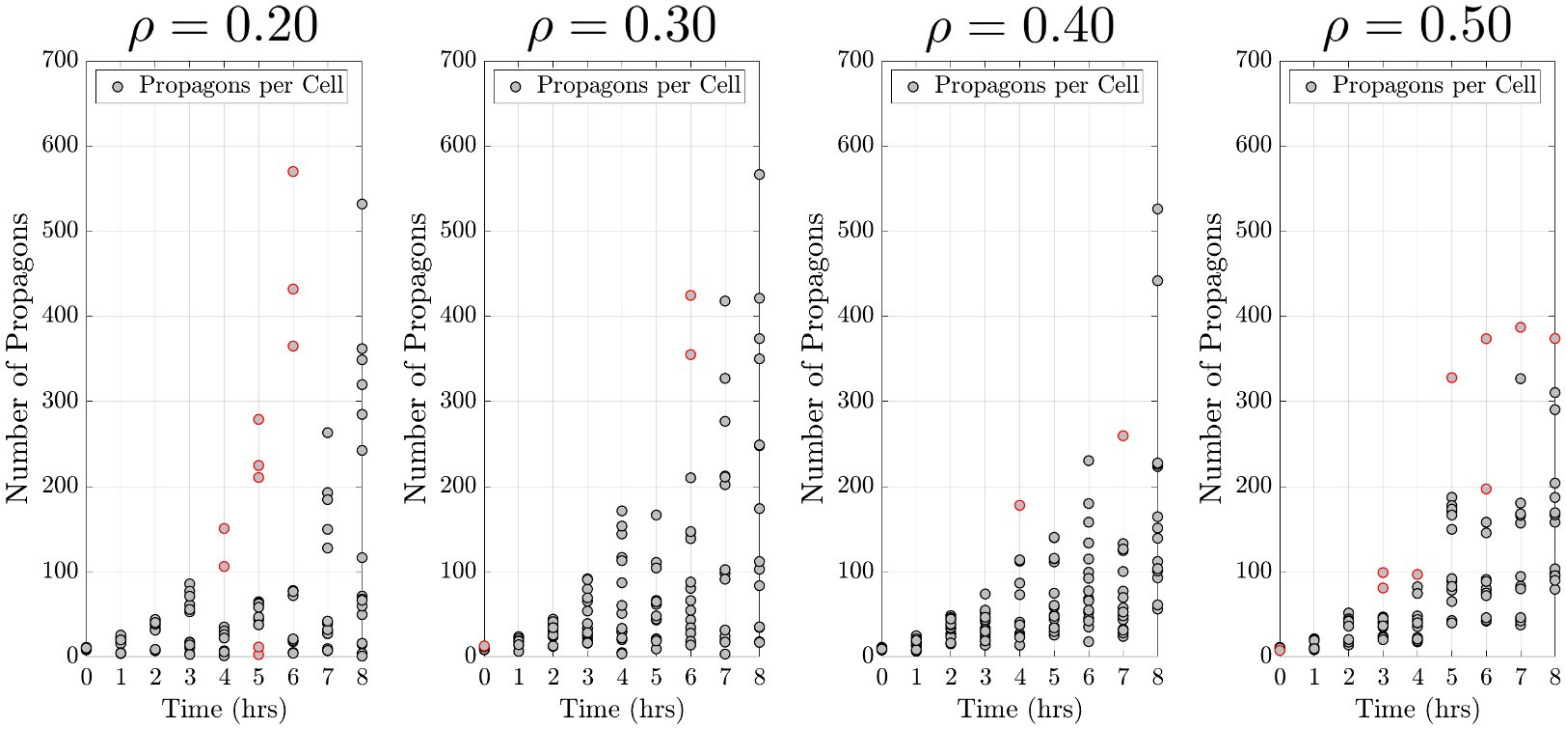
Simulated data. The simulated data was generated using a replication rate of λ = 0.70 hr^−1^ and four different transmission biases (*ρ*). Samples were generated per experimental hour at a rate of 16 samples per hour. Data points outlined in red were determined to be outliers by the IQR method.

In our investigation we considered the effects of replication rates, transmission biases, and sampling rates on our ability of recover known kinetic parameter values. Fig 3 shows four examples of simulated data produced by rejection sampling in the cases of four intracellular constituents biases *ρ* = 0.20, 0.30, 0.40, 0.50, a replication rate of λ = 0.70 hr^−1^, and a sampling rate of 16 samples per hour. The noise in the simulated data presented in Fig 3 is a consequence of simulating data with rejection sampling from the distribution of propagons over time in the ATP model.

Parameter estimations are made using the likelihood formulation and the AM algorithm 1 outlined in the methods section. We generate simulated data with three sampling rates: 8, 16, and 32 samples per hour, with replication rates λ = 0.5, 0.7, 0.9 (hr^−1^) and transmission bias *ρ* = 0.2, 0.3, 0.4, 0.5, for 36 total possible combinations. The sampling rates were chosen to reflect the experimental data which contain approximately 16 samples per hour per dataset. To robustly assess the effects of the sampling rates on our ability to recover known parameter choices, we tested our methods on 500 generated datasets for each of the 36 sampling rate and parameter combinations. The AM algorithm 1 was applied to each of the 500 simulated datasets and each resulting AM chain iteration is used to compute a mean parameter estimate. This results in 500 singleton parameter estimates for each of the two parameters. We use these 500 singleton parameter estimates to form a 95% credible interval for each of the 36 parameter and sampling rate combinations. We opted not to store each of the 500 AM chain iterations and combine them to form the credible intervals due to the burden on storage capacity. We present these results in Table 1.

**Table 1:** Credible intervals (95%) for parameter estimates on simulated data. The table summarizes the parameter inference results for twelve (λ, *ρ*) parameter pairs and three sampling rates using data simulated from the ATP model.

*θ*		8 Samples/Hour		16 Samples/Hour		32 Samples/Hour	
λ	*ρ*	λ	*ρ*	λ	*ρ*	λ	*ρ*
0.5	0.2	(0.49,0.52)	(0.19,0.21)	(0.50,0.52)	(0.19,0.20)	(0.50,0.51)	(0.20,0.20)
	0.3	(0.49,0.53)	(0.28,0.32)	(0.49,0.51)	(0.29,0.31)	(0.50,0.51)	(0.30,0.31)
	0.4	(0.49,0.51)	(0.38,0.42)	(0.50,0.51)	(0.39,0.41)	(0.50,0.51)	(0.39,0.41)
	0.5	(0.49,0.50)	(0.48,0.49)	(0.50,0.50)	(0.49,0.50)	(0.50,0.50)	(0.49,0.50)
0.7	0.2	(0.69,0.72)	(0.19,0.21)	(0.70,0.72)	(0.19,0.21)	(0.70,0.71)	(0.20,0.20)
	0.3	(0.69,0.73)	(0.28,0.32)	(0.69,0.71)	(0.29,0.31)	(0.69,0.71)	(0.30,0.31)
	0.4	(0.69,0.72)	(0.38,0.42)	(0.70,0.71)	(0.39,0.41)	(0.70,0.71)	(0.39,0.41)
	0.5	(0.69,0.70)	(0.48,0.49)	(0.70,0.70)	(0.48,0.50)	(0.70,0.70)	(0.49,0.50)
0.9	0.2	(0.89,0.92)	(0.19,0.21)	(0.90,0.92)	(0.19,0.21)	(0.90,0.91)	(0.20,0.20)
	0.3	(0.89,0.93)	(0.29,0.32)	(0.89,0.91)	(0.29,0.31)	(0.90,0.91)	(0.30,0.31)
	0.4	(0.89,0.92)	(0.39,0.42)	(0.90,0.91)	(0.39,0.41)	(0.90,0.91)	(0.39,0.41)
	0.5	(0.89,0.91)	(0.48,0.49)	(0.90,0.90)	(0.48,0.50)	(0.90,0.90)	(0.49,0.50)

In all cases we found that we can successfully detect differences in replication rates and asymmetric transmission biases. As expected, we observed that increasing the sampling rate led to more precise parameter estimates. In the cases where we estimate a symmetric transmission bias (*ρ* = 0.5), the credible intervals never capture this value. That is due to the fact that we estimate *ρ* in the interval [0, 0.5] because of the symmetry in our model about the point *ρ* = 0.5 in the interval [0, 1]. However, in Table 1 we see that as we increase the sampling rate, the estimates get closer to the true value of *ρ* = 0.5. In the experimental data we are interested in removing outliers using the interquartile range (IQR) method [33], so we studied the effects of removing possible “outliers” or extreme values using this method from our simulated data to study the effects on our ability to recover the true parameter values. We found that removing such outliers from our simulated data led to a slight improvement in our ability to recover the true parameter values (see Table F in S1 Text).

### Parameter inference on experimental data

**Fig 4:**
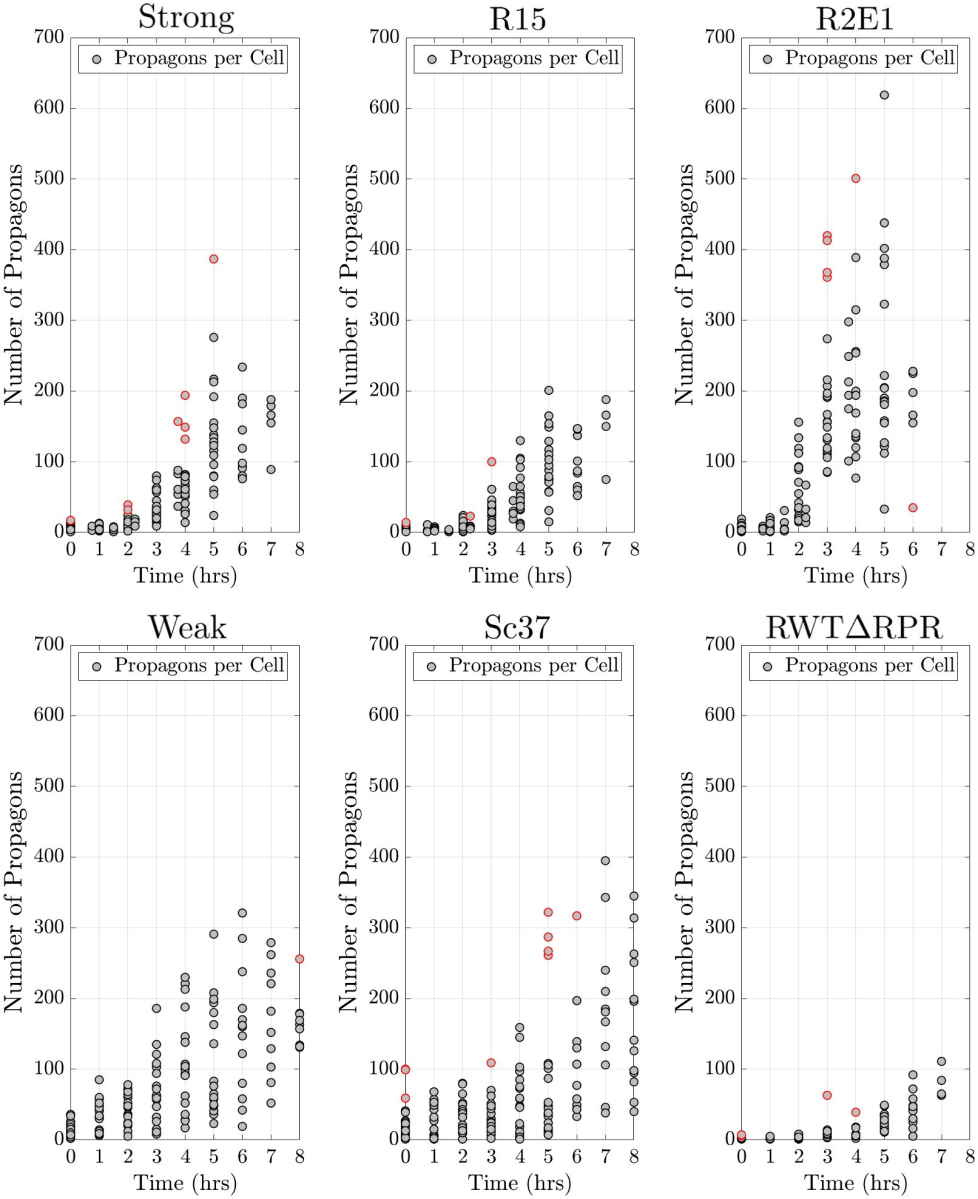
Experimental propagon counts for six prion variants. This experimental data was obtained through propagon recovery experiments (see the biological background section for more details). Data points outlined in red were determined to be outliers by the IQR method.

After verifying that our methods allows us to recover known replication rate and transmission bias parameters from simulated datasets, we apply our methods to experimental data from six prion variants. We consider the propagon replication experiments for six Sup35 variants that have alternative folds, aggregate to form propagons, and transmit the [*PSI*^+^] phenotype: Weak, Sc37, Strong [34], RWTΔRPR, R15, and R2E1 [12]. The experimental results from propagon recovery assays for the six prion variants are presented in Fig 4. Note that all variants exhibit heteroscedasticity in the number of propagons in time.

We consider two variations of our model for the division of propagons among dividing yeast cells. First we consider the model *Z*_*S*_ ≔ *Z*_*S*_(*t*, *a*; λ, *ρ* = 0.5), symmetric division of propagons between dividing cells where our model collapses to the model first proposed by [35] and we estimate the replication rate λ and fix the transmission bias at *ρ* = 0.5. Secondly we consider the model *Z*_*A*_ ≔ *Z*_*A*_(*t*, *a*; λ, *ρ*), asymmetric transmission of propagons where we estimate both the propagon replication rate λ and the propagon transmission bias *ρ*.

The general formulation of our likelihood also allows us to consider the possibility that the start of the exponential phase of prion amplification begins at a time after the start of the experiment (Fig 4). For this, application of the AM algorithm on the experimental data ${\left\{a\left(t_{i}\right)\right\}}_{i=1}^{m}$ is performed by using the data where *t*_*i*_ ≥ *T*_Δ_ for *T*_Δ_ hours into the experiment (see Tables A-E in S1 Text). Then we apply model selection to determine *T*_Δ_, the point of time into the experiment that best describes the beginning of the exponential phase of propagon replication.

In Table 2, we present the replication rate, transmission bias, the start of exponential growth phase, and the percent model weight for the model, *Z*_*A*_ or *Z*_*S*_, that best explains each dataset. Through our analysis we found that a model which includes the cell replication rate and asymmetric transmission of propagons during cell division (*Z*_*A*_), best explains the experimental propagon recovery data for all prion variants (model weights > 99%, Table 2). In the case of the Weak prion variant, we found that the dataset where outliers were kept and an asymmetric division model, *Z*_*A*_, best explained the propagon dynamics for the Weak variant with a model weight of 99.34%. For each variant we detected differences in the replication rates and transmission bias. Across the six prion variants we detected propagon transmission biases, the proportion of propagons transmitted to daughter cells, to be between 22% and 38%, *ρ* ∈ (0.22, 0.38). For the three prion variants RWTΔRPR, Strong, and R15, we found a delay in the start of the exponential growth phase of propagon replication. RWTΔRPR was found to have the largest delay (*T*_Δ_ = 2 hrs) before the beginning of the exponential growth phase, which was identified from experimental data without the outliers identified through the IQR method. Without the work to detect *T*_Δ_, we underestimate the replication rate for this variant (see Tables C, G, and H in S1 Text).

**Table 2:** Parameter estimates and credible intervals (95%) for six prion variants. The column labeled *T*_Δ_ indicates the point of time into the experiment that best describes the beginning of the exponential phase of propagon replication. The column labeled %W presents the percent model weight for *Z*_*A*_, the model for asymmetric division of propagons. The asterisk (*) indicates the dataset not filtered for outliers (raw data) was selected using AIC^*c*^.

Prion Variant	Replication Rate (λ, hr^−1^)	Transmission Bias (*ρ*, proportion)	*T*_Δ_ (hrs)	%W	Model
Weak	0.88(0.87,0.88)	0.27(0.26,0.27)	0*	99.34	*Z*_A_*
Sc37	0.81(0.79,0.82)	0.23(0.22,0.23)	0	100.0	*Z*_*A*_
RWTΔRPR	0.77(0.72,0.80)	0.31(0.29,0.33)	2	100.0	*Z*_*A*_
Strong	1.13(1.11,1.16)	0.36(0.34,0.38)	0.75	100.0	*Z*_*A*_
R15	1.14(1.10,1.18)	0.32(0.29,0.35)	0.75	100.0	*Z*_*A*_
R2E1	1.25(1.22,1.29)	0.29(0.26,0.33)	0	100.0	*Z*_*A*_

The parameter estimates, corrected AIC, percent model weights, and the postprocessing of the Metropolis chain iterations for all the cases considered are summarized in the methods section.

## Discussion and conclusion

In this work we presented a structured population model that generalizes the work presented in [13, 35], to study propagon replication dynamics and the bias in their transmission among proliferating yeast cells. We developed an inverse problem formulation that consists of an interpretable likelihood formulation descriptive of the propagon recovery experiment. We first verified that we could recover known propagon replication parameters from simulated data, then used our inverse problem formulation to study propagon replication in six prion variants. Additionally, the likelihood formulation allowed us to consider the presence of influential outliers and if there was a delay in observing the exponential phase of propagon replication in the experimental data.

We were able to detect differences in propagon replication rates and bias in their transmission during cell division among the six prion variants that we studied. We found that the Weak prion variant had a lower replication rate and transmission bias, λ = 0.88 hr^−1^ and *ρ* = 0.27 respectively, when compared to the Strong prion variant which had a higher replication rate and transmission bias, λ = 1.13 hr^−1^ and *ρ* = 0.36 respectively. These findings are consistent with previous studies of propagon size [7, 36], and fragmentation rates [8, 25] of Weak and Strong propagons, where Weak propagons are larger and replicate at a lower rate than Strong propagons, thus it would be reasonable to observe a higher rate of propagon replication and a more significant transmission bias in Strong than in Weak.

In this work we detected differences in transmission biases for each of the six prion variants, however the reason for these differences cannot be found with our current model formulation. We would expect the transmission bias to be proportional to the volume of the daughter cell to that of the mother and daughter volumes or *ρ*_1_ ≈ *V*_*D*_/(*V*_*M*_ + *V*_*D*_) ≈ 40%, and *ρ*_2_ ≈ *V*_*M*_/(*V*_*M*_ + *V*_*D*_) ≈ 60%, as has been found in [15] for different prion variants not considered in this work. From Table 3 adapted from [37], these ratios are generation dependent. However, our estimates for the transmission biases remain close to these generational dependent values. Therefore, it is possible that the differences in transmission biases we are capturing come from these generation based volume differences. In future models, we will need to incorporate generation based volume differences to discern differences in transmission biases from generation based daughter cell volume.

**Table 3:** Relative volume of daughter cells by generation. Where *V*_*M*_ is the mother cell volume and *V*_*D*_ is the daughter volume. This table was adapted from Table 2 in [37].

Generation	1	2	3	4
*V*_*D*_/(*V*_*D*_ + *V*_*M*_)	0.40	0.32	0.29	0.25

To our knowledge our work is the first to use propagon amplification assays and a structured population model (ATP model) to recover both the propagon replication rate and the transmission bias during cell division. The work in [13] uses propagon amplification assays and a structured population model that assumes symmetric transmission of propagons between dividing cells to determine propagon replication rates for two yeast variants but did not directly address the asymmetric bias in propagon transmission during cell division. Separate work presented in [15] has used curing experiments, where the application of GdnHCl causes inhibition of propagon replication and leads to elimination of the [*PSI*^+^] phenotype through dilution by cell division and a model that captures asymmetric cell division through unequal transmission of propagons through cell division [38], to recover the propagon transmission bias. They considered the propagon transmission bias as the probability of propagon transmission to a budding daughter cell in their work. The curing experiment eliminates [*PSI*^+^] phenotype and as such, a propagon replication rate cannot be computed. We note that our model can also be used to calculate the asymmetric transmission bias using data from curing experiments by setting the replication rate equal to zero (λ = 0 hr^−1^). The number of propagons will decrease through asymmetric transmission of propagons between dividing cells.

In this work we detail the importance of the propagon replication rate λ, the asymmetric propagon transmission bias *ρ* and detection of a delay *T*_Δ_, before observing the exponential growth phase of prion replication, to explain prion replication in a proliferating yeast cell colony. In future work we intend to build on this framework by incorporating cell maturation, a state during which cells can grow but cannot divide until they are fully mature. We have made an effort to be clear about the difference between *asymmetric cell division* and *asymmetric transmission of intracellular constituents*, but in future models we plan to incorporate cell volume into our models, so that we can distinguish between effects due to volume and those due to transmission bias. Also, in this work our intracellular propagon replication model assumed exponential growth because a clear steady state, or carrying capacity, was not observed in the experimental data, but perhaps by running the propagon amplification assays over a longer period of time we would begin to observe such a steady state. This would require the application of a more complex intracellular model to capture such a carrying capacity and would require methods such as those presented in [39] to numerically solve such a propagon replication model. As previously mentioned, another possible application with our modeling framework that we have not yet considered is the possibility of its application to study curing experiments.

## Methods

In this section we begin by presenting our model, a system of partial differential equations (PDEs) for the intracellular process of propagon replication and their transmission through the cellular process of division. We present intermediate quantities that allow the decoupling of the PDE system and present the explicit solutions to the model that we consider in this work. Then we show that the intermediate quantities used to derive explicit solutions facilitate a likelihood formulation for parameter estimation and model selection. We describe how to generate simulated data with the model solutions and that we can recover the true kinetic parameters with our likelihood formulation and our implementation of the adaptive Metropolis (AM) algorithm. We conclude this section by detailing how we overcome numerical issues with the implementation of our likelihood formulation.

### Asymmetric transmission of propagons model

We seek to model the number of propagons or a single prion variant in a population of actively dividing cells. Let *a*(*t*) be the number of propagons a cell has *t* hours after dividing, then

$$\begin{array}{c} \frac{da}{dt}=\eta \left(a;\theta \right) \end{array}$$ (1)

where *η* is the intracellular propagon amplification model that depends on the current number of propagons *a* and ***θ***, the kinetic parameter(s) that govern the propagon replication and transmission dynamics (i.e. replication rate and transmission bias). We model the propagon distribution dynamics in the yeast cell population *Y*(*t*, *a*), as evolving in time according to the transport equation:

$$\begin{array}{c} \frac{\partial }{\partial t}Y\left(t,a\right)+\frac{\partial }{\partial a}\left(\eta \left(a;\theta \right)Y\left(t,a\right)\right)=0. \end{array}$$ (2)

**Fig 5:**
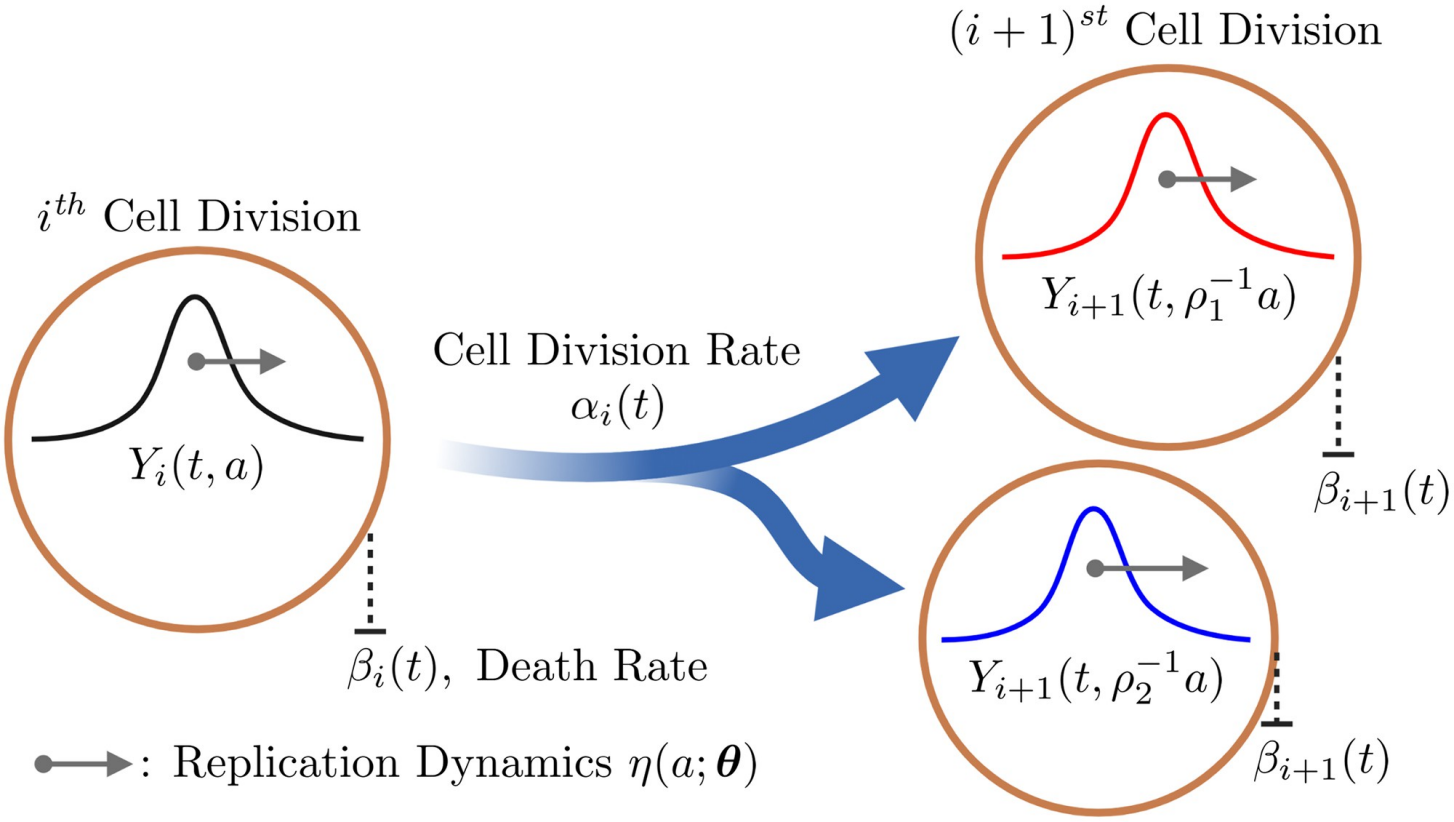
Asymmetric transmission of propagons model schematic. The model dynamics of intracellular propagon replication and cell division from generation *i* to generation *i* + 1. The black arrow (→) illustrates the intracellular increase in the number of propagons over time. The remaining parameters are detailed in the methods.

However, we are interested in tracking *Y*_*i*_(*t*, *a*), the distribution of propagons in cells that have undergone *i* divisions, *t* hours since the start of the experiment (see Fig 5). These propagon dynamics in the population of dividing cells are captured by the following system of *M* + 1 coupled PDEs, which we refer to as the Asymmetric Transmission of Propagons (ATP) model:

$$\begin{array}{c} \begin{array}{cc} \frac{\partial }{\partial t}Y_{0}\left(t,a\right)+\frac{\partial }{\partial a}\left(\eta \left(a;\theta \right)Y_{0}\left(t,a\right)\right) & =-\left(\alpha_{0}\left(t\right)+\beta_{0}\left(t\right)\right)Y_{0}\left(t,a\right),\\ \frac{\partial }{\partial t}Y_{1}\left(t,a\right)+\frac{\partial }{\partial a}\left(\eta \left(a;\theta \right)Y_{1}\left(t,a\right)\right) & =-\left(\alpha_{1}\left(t\right)+\beta_{1}\left(t\right)\right)Y_{1}\left(t,a\right)+D_{1}\left(t,a\right),\\ & \vdots \\ \frac{\partial }{\partial t}Y_{i}\left(t,a\right)+\frac{\partial }{\partial a}\left(\eta \left(a;\theta \right)Y_{i}\left(t,a\right)\right) & =-\left(\alpha_{i}\left(t\right)+\beta_{i}\left(t\right)\right)Y_{i}\left(t,a\right)+D_{i}\left(t,a\right),\\ & \vdots \\ \frac{\partial }{\partial t}Y_{M}\left(t,a\right)+\frac{\partial }{\partial a}\left(\eta \left(a;\theta \right)Y_{M}\left(t,a\right)\right) & =-\left(\alpha_{M}\left(t\right)+\beta_{M}\left(t\right)\right)Y_{M}\left(t,a\right)+D_{M}\left(t,a\right). \end{array} \end{array}$$ (3)

The right hand side of each PDE captures the rate of cells lost in that generation from cell division and cell death, and accounts for new cells from cell division in the previous generation. Here *α*_*i*_(*t*) is the rate of cell division, and *β*_*i*_(*t*) the rate of cell death in the *i*^*th*^ generation. The rate of increase in the number of cells in the *i*^th^ generation is represented by the term

$$\begin{array}{c} D_{i}\left(t,a\right)=\rho_{1}^{-1}\alpha_{i-1}\left(t\right)Y_{i-1}\left(t,\rho_{1}^{-1}a\right)+\rho_{2}^{-1}\alpha_{i-1}\left(t\right)Y_{i-1}\left(t,\rho_{2}^{-1}a\right), \end{array}$$ (4)

where *ρ*_1_ and *ρ*_2_ control the transmission of propagons between dividing cells. To maintain the conservation of propagons during division we require *ρ*_1_ + *ρ*_2_ = 1, showing only one degree of freedom or that if *ρ*_1_ = *ρ* ∈ (0, 1), then *ρ*_2_ = 1 − *ρ*. Note that letting *ρ*_1_ = *ρ*_2_ = 1/*γ* reduces our model to that proposed by [35]. Also, note that this formulation implies that for conservation and symmetric transmission of propagons during cell division *ρ* = 1/*γ* = 1/2. The work by [40] first generalized the work by [35] to study T cells traced with carboxyfluorescein diacetate succinimidyl ester to include their resting and cycling phases (delay in division) while considering an asymmetric division with a system of delay hyperbolic PDEs. However, in our modeling framework because of the duration of the experiment (8 hrs), we expect most cell divisions to be the product of mature cells actively producing daughter cells so we do not consider synchronous delays in division.

In this work, we are interested in determining the effect of *ρ*, the propagon transmission biased between dividing cells. To this end, we can bound the number of cell divisions (*M*) because the duration of the experiment of interest is finite. We bound the maximum number of cell divisions up to *M* = 6 generations because yeast cells divide every 1.5 hours and the longest duration of the propagon recovery experiments considered in this work is eight hours. Then to solve the ATP model, Eq (3), we must specify initial distributions for each generation

$$\begin{array}{c} Y_{0}\left(0,a\right)=ϒ\left(a\right)\;\text{and}\;Y_{i}\left(0,a\right)=0\;\text{for}\;\text{all}\;i>0. \end{array}$$ (5)

Where ϒ(*a*), is the initial intracellular distribution of propagons at the start of the experiment.

### Analytic solutions and model decomposition

The formulation of the ATP model, Eqs (3)–(5) presented in the methods, allows for computation of intermediate quantities that allow for the decoupling of population and intracellular propagon dynamics in Eq (3). First, the total number of cells resulting from the *i*^*th*^ division, since the beginning of the experiment, at time *t* is given by

$$\begin{array}{c} n_{i}\left(t\right)=\int_{R}Y_{i}\left(t,a\right)da, \end{array}$$ (6)

where *R* depends on the intracellular propagon amplification dynamics model in Eq (1), and is taken to be *R* = (0, ∞) in our work (see Corollary 1). The normalized density of propagons can then be defined by

$$\begin{array}{c} y_{i}\left(t,a\right)=\frac{Y_{i}\left(t,a\right)}{n_{i}\left(t\right)},\;\text{for}\;n_{i}\left(t\right)>0, \end{array}$$ (7)

and *y*_*i*_(*t*, *a*) = 0 otherwise. This quantity represents the intracellular dynamics of the dividing cells in our population of interest. We define the initial number of cells at the start of the experiment by

$$\begin{array}{c} N_{0}=\int_{R}Y_{0}\left(0,a\right)da, \end{array}$$ (8)

and the initial normalized propagon density

$$\begin{array}{c} y_{0}\left(0,a\right)=\frac{Y_{0}\left(0,a\right)}{N_{0}}. \end{array}$$ (9)

Using the quantities defined above and Eq (3), the following theorem holds for Eq (1) defined by an exponential growth model *η*(*a*; ***θ***) = λ*a*, where *a* ∈ *R* = (0, ∞).

**Theorem 1**. *The solution of the system defined by* Eqs (3) *and* (4), *with initial conditions given by* Eq (5) *is given by*:

$$\begin{array}{c} Y_{i}\left(t,a\right)=n_{i}\left(t\right)y_{i}\left(t,a\right),\;for\;0\leq i\leq M, \end{array}$$ (10)

*in which*:

1. *n*_*i*_(*t*) *is the solution of the system of ODEs*:
  $$\begin{array}{c} \begin{array}{cc} i=0: & \frac{dn_{0}}{dt}=-\left(\alpha_{0}\left(t\right)+\beta_{0}\left(t\right)\right)n_{0},\\ for\;1\leq i\leq M: & \frac{dn_{i}}{dt}=-\left(\alpha_{i}\left(t\right)+\beta_{i}\left(t\right)\right)n_{i}+2\alpha_{i-1}\left(t\right)n_{i-1}, \end{array} \end{array}$$ (11)
  *where n*_0_(0) = *N*_0_, *and n*_*i*_(0) = 0 *for i* ≥ 1.
2. *y*_*i*_(*t*, *a*) *is the solution to the PDE*
  $$\begin{array}{c} \frac{\partial y_{i}\left(t,a\right)}{\partial t}+\frac{\partial (\eta \left(a;\theta \right)y_{i}\left(t,a\right))}{\partial a}=0 \end{array}$$ (12)
  *with initial conditions* yi(0,a)≔(12)i∑k=0i(ik)ρ1k-iρ2-kϒ(ρ1k-iρ2-ka), *and*
3. *the solution y*_*i*_(*t*, *a*) *satisfies the recursive property*
  $$\begin{array}{c} y_{i}\left(t,a\right)=\frac{1}{2}(\rho_{1}^{-1}y_{i-1}\left(t,\rho_{1}^{-1}a\right)+\rho_{2}^{-1}y_{i-1}\left(t,\rho_{2}^{-1}a\right)), \end{array}$$ (13)
  *for all* 0 ≤ *i* ≤ *M*.

Unlike the theorems presented in [35] and [40], in Theorem 1 we highlight the fact that *y*_*i*_(*t*, *a*) must satisfy Eqs (12) and (13), in order for Eq (10) to be a solution to the system defined by Eq (3). This is to highlight that for non-linear intracellular dynamics models (Eq (1)), the property given by Eq (13) may no longer be satisfied [39]. The proof of Theorem 1 follows the structure of the proof in [35], therefore we simply outline the proof of Theorem 1 here.

*Proof*. First, we substitute the *Y*_*i*_(*t*, *a*) terms in Eq (3) with the decomposition given by Eq (10). Then we simplifying the terms of Eq (3), and using Eqs (11) and (12) leads to the recursive expression in Eq (13) completing the proof.

In this work we are modeling propagon replication where at the beginning of the experiment the initial condition is that of a distribution of low propagon counts in the yeast cell population as observed in the propagon recovery data (see Fig 4). This initial low number of propagons is followed by a period of exponential growth where a steady state in the number of propagons is not yet observed. Corollary 1 captures this phase of growth, where we assume that the rate of propagon replication is proportional to the current number of propagons present within a yeast cell. This type of propagon proliferation assumes that there is an unlimited amount of normally folded (soluble) protein that can be alternatively folded and lead to the continued formation of prion propagons.

**Corollary 1**. *The solution of the system defined by* Eqs (3) *and* (4) *with an intracellular propagon replication model η*(*t*, *a*) = λ*a, is*

Yi(t,a)=ni(t)∑k=0i(ik)ρ1k-iρ2-kexp(-λt)ϒ(ρ1k-iρ2-kaexp(-λt))for0≤i≤M. (14)

*Where n*_*i*_(*t*) *is the solution to* Eq (11).

*Proof*. Following [40], we solve Eq (12) via the method of characteristics. This yields

yi(t,a)=(12)i∑k=0i(ik)ρ1k-iρ2-kexp(-λt)ϒ(ρ1k-iρ2-kaexp(-λt))for0≤i≤M.

To show that *y*_*i*_(*t*, *a*) satisfies the recursive property in Eq 13 we prove the equivalent expression $y_{i+1}\left(t,a\right)=\frac{1}{2}(\rho_{1}^{-1}y_{i}\left(t,\rho_{1}^{-1}a\right)+\rho_{2}^{-1}y_{i}\left(t,\rho_{2}^{-1}a\right))$ holds, see S1 Text. Then, replacing *y*_*i*_(*t*, *a*) in Eq (10), and inserting this result in Eq (3) proves Corollary 1.

The following corollary presented in [35] involves the solution of Eq (11) under a special case of constant cell division rate *α*_*i*_(*t*) = *α* and constant cell death *β*_*i*_(*t*) = *β*. In [35] the authors consider *β*_*i*_(*t*) = *β* > 0, but the solution also holds for *β* = 0, which is the assumption in this work.

**Corollary 2**. *Let α*_*i*_(*t*) = *α* ≥ 0 *and β*_*i*_(*t*) = *β* ≥ 0, *for all* 0 ≤ *i* ≤ *M*, *the solution to* Eq (11) *is*

$$\begin{array}{c} n_{i}\left(t\right)=\frac{{\left(2\alpha t\right)}^{i}}{i!}\text{exp}\left(-\left(\alpha +\beta \right)t\right)N_{0},\;for\;0\leq i\leq M. \end{array}$$ (15)

*Proof*. Straightforward by plugging Eq (15) into Eq (11).

### Likelihood problem formulation and model selection

In this work we are interested in estimating the kinetic parameters of our model, Eqs (3)–(5) with the propagon amplification models presented in the methods, using experimental data. We are interested in the probability of the observed data for a given value of the kinetic parameters ***θ*** of the parameter space **Θ**, denoted *L*(***θ***|*Data*). Let $N\left(t\right)=\sum_{i=0}^{M}n_{i}\left(t\right)$, the total number of cells at time *t*, $Z\left(t,a;\theta \right)=\sum_{i=0}^{M}Y_{i}\left(t,a\right)$ represent the total yeast cell population propagon density after *M* cell divisions, and ***θ*** represent the kinetic parameters. Further, let the experimental observations *a*_*k*_ observed at time *t*_*k*_ consisting of propagon counts be ${\left\{\left(a_{k},t_{k}\right)\right\}}_{k=1}^{m}$ (see the Results section). Then, the likelihood of the kinetic parameters ***θ***, given the propagon data is defined as follows

$$\begin{array}{c} \begin{array}{cc} L(\theta |Data) & =\prod_{k=1}^{m}P\left(\left\{t_{k},a_{k}\right\};\theta \right),\\ & =\prod_{k=1}^{m}\{\sum_{i=0}^{M}[(\frac{n_{i}\left(t_{k}\right)}{N(t_{k})})y_{i}\left(t_{k},a_{k};\theta \right)]\},\\ & =\prod_{k=1}^{m}\sum_{i=0}^{M}\frac{Y_{i}\left(t_{k},a_{k};\theta \right)}{N(t_{k})},\\ & =\prod_{k=1}^{m}\frac{Z(t_{k},a_{k};\theta )}{N(t_{k})}. \end{array} \end{array}$$ (16)

where *P*({*t*_*k*_, *a*_*k*_}; ***θ***) is the probability of observing *a*_*k*_ propagons at time *t*_*k*_ given model parameters ***θ***. This probability is the product of $\frac{n_{i}\left(t_{k}\right)}{N(t_{k})}$, the probability that the observation *a*_*k*_ came from a cell in the *i*^*th*^ cell division and *y*_*i*_(*t*_*k*_, *a*_*k*_; ***θ***), the probability that the cell in the *i*^*th*^ cell division at time *t*_*k*_ contains *a*_*k*_ propagons. The product of both terms is summed over the number of divisions that can be observed during the experiment. This is because a cell at any point in the experiment results from a finite number of cell divisions since the beginning of the experiment.

In this work we are interested in considering two mathematical modeling scenarios. The first *Z*_*S*_(*t*, *a*; λ, *ρ* = 0.5), symmetric cell division where we estimate the posterior distribution of the replication rate (λ) and fix the transmission bias at *ρ* = 0.5, and the second *Z*_*A*_(*t*, *a*; λ, *ρ*), asymmetric cell division where we estimate the posterior distributions of the replication rate λ and the posterior distribution of the propagon transmission bias *ρ*. With the likelihood formulation we compute the Akaike Information Criterion (AIC)

$$\text{AIC}=-2\cdot \text{log}(L(\hat{\theta }|Data))+2K,$$

where $\hat{\theta }$ are the mean parameter estimates that best explain the data and *K* is the number of free parameters [41]. To account for bias due to different number of parameters and data sizes, we use the AIC bias correction

$${\text{AIC}}^{c}=\text{AIC}+\frac{2K(K+1)}{m-K-1},$$

where *m* is the sample size [42]. These AIC^*c*^ values are used to compute the AIC^*c*^ difference

$$\Delta_{i}=\text{AIC}{}_{i}^{c}-\text{min}\left({\text{AIC}}^{c}\right),$$

for the *i*^*th*^ model and prion variant data. Finally, we can compute the relative model weights

$${\text{W}}_{i}=\frac{\text{exp}(-\frac{1}{2}\Delta_{i})}{\sum_{m=1}^{M}\text{exp}\left(-\frac{1}{2}\Delta_{m}\right)},$$

of each model [43] with each prion variant dataset. We interpret this quantity as the probability that a model is the best approximation to the replication and division of propagons during cellular proliferation given the experimental data.

### Adaptive metropolis algorithm

In this work we follow the procedure described in [44], a Metropolis algorithm with an adaptive Metropolis (AM) step to estimate the target distribution *π*(***θ***), with *p* kinetic parameter variables **Θ**. Using an initial value for each kinetic parameter ***θ***_0_, as the starting condition, a random candidate ***θ***_*new*_ is drawn from a proposal distribution *J* of the parameters ***θ***. Thus ***θ***_*new*_ ∼ *J*(***θ***_*new*_|***θ***_*i*−1_), is drawn in every iteration.

The target distribution *π*(***θ***) is given using previously defined likelihood function *L*(***θ***|*Data*),

$$\begin{array}{c} \pi \left(\theta \right)=\frac{L\left(\theta |Data\right)\pi_{0}\left(\theta \right)}{\pi (Data)}, \end{array}$$ (17)

where the random parameter variables **Θ** have a known and possibly uninformative prior density *π*_0_(***θ***), $\pi \left(Data\right)=\int_{R^{p}}L\left(\theta |Data\right)\pi_{0}\left(\theta \right)d\theta$, and *p* is the dimension of the parameter set. In this work we choose the noninformative prior *π*_0_(***θ***) = *U*(0, 1)^*p*^ and *J*(***θ***_*new*_|***θ***_*i*−1_) = *N*(***θ***_*i*−1_; *V*) to be normally distributed with covariance matrix *V*. The acceptance probability then follows

$$\begin{array}{c} \alpha \left(\theta_{new}|\theta_{i-1}\right)=\text{min}\{1,\frac{\pi \left(\theta_{new}\right)J\left(\theta_{i-1}|\theta_{new}\right)}{\pi \left(\theta_{i-1}\right)J\left(\theta_{new}|\theta_{i-1}\right)}\}=\text{min}\{1,\frac{L(\theta_{new}|Data)}{L(\theta_{i-1}|Data)}\}. \end{array}$$ (18)

In Eq (18), the terms involving *J* cancel because by design *J* is a symmetric proposal distribution. Now, with probability *α*(***θ***_*new*_|***θ***_*i*−1_), we accept ***θ***_*new*_, and set ***θ***_*i*_ ≔ ***θ***_*new*_. Otherwise, with probability 1 − *α*(***θ***_*new*_|***θ***_*i*−1_), we set ***θ***_*i*_ ≔ ***θ***_*i*−1_.

Following [45], the adaptive step is preceded by a non-adaptive period of length *k* where ***θ***_1_, ***θ***_2_, ⋯, ***θ***_*k*_ are computed using an initial covariant matrix *V*_0_ = *V*. Following the non-adaptive step, the covariance matrix is computed using the previous chain values

$$\begin{array}{c} V_{i}=s_{p}\text{cov}(\theta_{0},\theta_{1},\ldots ,\theta_{i})+\varepsilon I_{p},\;\text{for}\;i\geq k. \end{array}$$ (19)

Here *s*_*p*_ is a design parameter that depends on *p*. The term *εI*_*p*_ consists of the *p*-dimensional identity matrix and *ε* ≥ 0 to ensure that *V*_*i*_ remains positive definite. This formulation can quickly incur large computational cost, but this cost can be drastically reduced by use of the recursive update of the covariance [45]

$$\begin{array}{c} V_{i}=\frac{i-2}{i-1}V_{i-1}+\frac{s_{p}}{i-1}(\left(i-1\right){\hat{\theta }}_{i-2}{\hat{\theta }}_{i-2}^{T}-i{\hat{\theta }}_{\left(i-1\right)}{\hat{\theta }}_{\left(i-1\right)}^{T}+\theta_{\left(i-1\right)}\theta_{\left(i-1\right)}^{T}). \end{array}$$ (20)

In the application of the adaptive Metropolis algorithm to the simulated data and experimental data, we take *s*_*p*_ = 1.0, *ε* = 1 × 10^−6^, and

$$V_{0}=[\begin{array}{cc} \sigma_{\lambda \lambda }^{2} & \sigma_{\lambda \rho }^{2}\\ \sigma_{\rho \lambda }^{2} & \sigma_{\rho \rho }^{2} \end{array}],$$

where $\sigma_{\lambda \lambda }^{2}=0.1406$, $\sigma_{\lambda \rho }^{2}=\sigma_{\rho \lambda }^{2}=0$, and $\sigma_{\rho \rho }^{2}=0.0156$. A non-adaptive period length of *k* = 1500 for the simulated data and experimental data were established using Geweke’s convergence diagnostic as presented in [46], and performed a total of 2 × 10^6^ iterations. We avoid numerical issues in the direct evaluation of the likelihood formulation presented in Eq (16) by working with natural-logarithm version of the acceptance probability in Eq (18) using the numerically stable procedure outlined in the methods section.

To reduce the correlation among the AM estimates (***θ***), estimates after the burn-in period and after thinning at regular intervals of 50 iterations are used in our work. We use the integrated autocorrelation time (iac) as a measure of autocorrelation across the autocorrelation function (ACF) [47]. The chain iteration and final iacs are included in Figs A and B in S1 Text.

### Numerical implementation of the likelihood formulation

In our initial implementation of the likelihood formulation, we encountered significant numerical underflow, so we opted to work with the natural-logarithm form of the likelihood or log-likelihood. Recalling that $N\left(t\right)=\sum_{i=0}^{M}n_{i}\left(t\right)$ and that $Z\left(t\right)=\sum_{i=0}^{M}Y_{i}\left(t,a\right)$, taking the natural logarithm of Eq (16), we have

$$\begin{array}{c} \begin{array}{cc} \text{ln}\;L(\theta |Data) & =\sum_{k=1}^{m}\text{ln}\;(Z(t,a;\theta ))-\text{ln}\;(N(t)),\\ & =\sum_{k=1}^{m}\text{ln}\;(\sum_{i=0}^{M}Y_{i}\left(t_{k},a_{k};\theta \right))-\text{ln}(\sum_{i=0}^{M}n_{i}\left(t_{k}\right)). \end{array} \end{array}$$ (21)

Numerical evaluation of this log-likelihood is performed by generalizing the algebraic and numerically stable property ln(*a*_0_ + *a*_1_) = ln(*a*_0_) + ln(1 + exp(ln(*a*_1_) − ln(*a*_0_))) where ln(*a*_0_) > ln(*a*_1_). That is we evaluate $\text{ln}\;(\sum_{i=0}^{M}Y_{i}\left(t_{k},a_{k};\theta \right))$ by applying the natural log directly to each *i*^th^ analytic model solution *Y*_*i*_(*t*, *a*; ***θ***), then evaluating the resulting expression. The evaluation of $\text{ln}\;(\sum_{i=0}^{M}Y_{i}\left(t_{k},a_{k};\theta \right))$ is then found recursively using the ln (*Y*_*i*_(*t*_*k*_, *a*_*k*_; ***θ***)) terms as follows: let $\text{ln}\;q_{i}=\text{ln}\;Y_{i}\left(t,a;\theta \right){|}_{t=t_{k},a=a_{k}}$, and $q=\{\text{ln}\;q_{j}{\}}_{j=0}^{M}$ be the sorted values ln *q*_*i*_ in descending order, then $\text{ln}\;(\sum_{i=0}^{M}q_{i})$ is given by the numerically stable recursive function *ξ* as follows

$$\begin{array}{c} \xi (q)=\{\begin{array}{cc} q(1) & |q|=1\\ q(1)+\text{ln}(1+\text{exp}(\xi (q(∼1))-q(1))) & |q|>1 \end{array} \end{array}$$ (22)

where |⋅| is the cardinality or the number of elements in ***q*** and the notation “∼ 1” indicates all elements in ***q*** except the first. Implementing this formulation removed numerical underflow when evaluating the likelihood formulation (Eq (16)).

## Supporting information

S1 Text Recursive property to corollary 1, additional notes on parameter estimation, model selection, and experimental data. (PDF)
