# Supplementary material for: A structured model and likelihood approach to estimate yeast prion propagon replication rates and their asymmetric transmission
Source: PLoS Comput Biol. 2022 Jul 1;18(7):e1010107. doi: 10.1371/journal.pcbi.1010107 (PMC9249220; doi:10.1371/journal.pcbi.1010107)
Supplement: S1 Text — (PDF) [file pcbi.1010107.s001.pdf]

S1 Appendix

Recursive property to Corollary 1 in the main text

We show that the recursive property of Eq. (13), in Theorem 1 re-stated below in an equivalent form,

$$y_{i+1}(t,a) = \frac{1}{2}\left(\rho_1^{-1} y_i(t, \rho_1^{-1} a) + \rho_2^{-1} y_i(t, \rho_2^{-1} a)\right), \tag{1}$$

is satisfied by the solution

$$y_i(t,a) = \left(\frac{1}{2}\right)^i \sum_{k=0}^{i} \binom{i}{k} {\rho_1}^{k-i} {\rho_2}^{-k} \exp(-\lambda t) \Upsilon\left({\rho_1}^{k-i} {\rho_2}^{-k} a \exp(-\lambda t)\right),$$

in Corollary 1. That is, we show that the left hand side (LHS) of Eq. (1) is equal to the right hand side (RHS) of Eq. (1) with our solution. First we replace $y_i(t,a)$ in the RHS of the recursive equation with the solution from Corollary 1, resulting in ($s1$). We write out the first term ($k = 0$) from the first sum and the last term ($k = i$) in the second sum. The second sum is re-indexed with $k := k - 1$ and we combine the two sums, resulting in ($s2$). Using Pascal's rule

$$\binom{i}{k} + \binom{i}{k-1} = \binom{i+1}{k},$$

the equivalent expressions $\binom{i}{0} = \binom{i+1}{0}$ and $\binom{i}{i} = \binom{i+1}{i+1}$, results in ($s3$). The three terms can now be written as one sum ($s4$) which is the LHS of Eq. (1), or $y_{i+1}(t,a)$.

$$\begin{aligned}
\text{RHS} = \frac{1}{2}\Bigg(& \left(\frac{1}{2}\right)^i \sum_{k=0}^{i} \binom{i}{k} {\rho_1}^{k-i+1} {\rho_2}^{-k} \exp(-\lambda t) \Upsilon\left({\rho_1}^{k-i+1} {\rho_2}^{-k} a \exp(-\lambda t)\right) \\
& + \left(\frac{1}{2}\right)^i \sum_{k=0}^{i} \binom{i}{k} {\rho_1}^{k-i} {\rho_2}^{-k+1} \exp(-\lambda t) \Upsilon\left({\rho_1}^{k-i} {\rho_2}^{-k+1} a \exp(-\lambda t)\right) \Bigg), && (s1) \\
= \left(\frac{1}{2}\right)^{i+1} & \exp(-\lambda t) \Bigg(\binom{i}{0} \rho_1^{-i+1} \Upsilon\left({\rho_1}^{-i+1} a \exp(-\lambda t)\right) \\
& + \sum_{k=1}^{i} \left[\binom{i}{k} + \binom{i}{k-1}\right] \rho_1^{k-i+1} \rho_2^{-k} \Upsilon\left({\rho_1}^{k-i+1} {\rho_2}^{-k} a \exp(-\lambda t)\right) \\
& + \binom{i}{i} \rho_2^{i+1} \Upsilon\left({\rho_2}^{-i+1} a \exp(-\lambda t)\right) \Bigg), && (s2) \\
= \left(\frac{1}{2}\right)^{i+1} & \exp(-\lambda t) \Bigg(\binom{-i+1}{0} \rho_1^{-i+1} \Upsilon\left({\rho_1}^{-i+1} a \exp(-\lambda t)\right) \\
& + \sum_{k=1}^{i} \binom{i+1}{k} \rho_1^{k-i+1} \rho_2^{-k} \Upsilon\left({\rho_1}^{k-i+1} {\rho_2}^{-k} a \exp(-\lambda t)\right) \\
& + \binom{i+1}{i+1} \rho_2^{i+1} \Upsilon\left({\rho_2}^{-i+1} a \exp(-\lambda t)\right) \Bigg), && (s3) \\
= \left(\frac{1}{2}\right)^{i+1} & \sum_{k=0}^{i+1} \binom{i+1}{k} \rho_1^{k-i+1} \rho_2^{-k} \exp(-\lambda t) \Upsilon\left({\rho_1}^{k-i+1} {\rho_2}^{-k} a \exp(-\lambda t)\right), && (s4) \\
= y_{i+1}&(t,a), \\
= \text{LHS}&.
\end{aligned}$$

Adaptive metropolis algorithm

Figures A and B illustrate the chain iterations for the models and datasets selected after our model selection procedure. The appearance of increasing variance in the posterior estimates, as the number of iterations increases, is an artifact from plotting the AM algorithm chain iterations on a logscale. To reduce the correlation among the AM estimates ($\boldsymbol{\theta}$), estimates after the burn-in period and after thinning at regular intervals of 50 iterations are used in our work. In this work we use the integrated autocorrelation time (iac) as a measure of autocorrelation across the autocorrelation function (ACF), where an iac value of 1 indicates no autocorrelation. Note that there are no chain iteration and autocorrelation function figures for the division bias (ρ) under the model $Z_S(t, a; \lambda, \rho = 0.5)$ because in these cases the division bias parameter is fixed at $\rho = 0.5$ (symmetric transmission).

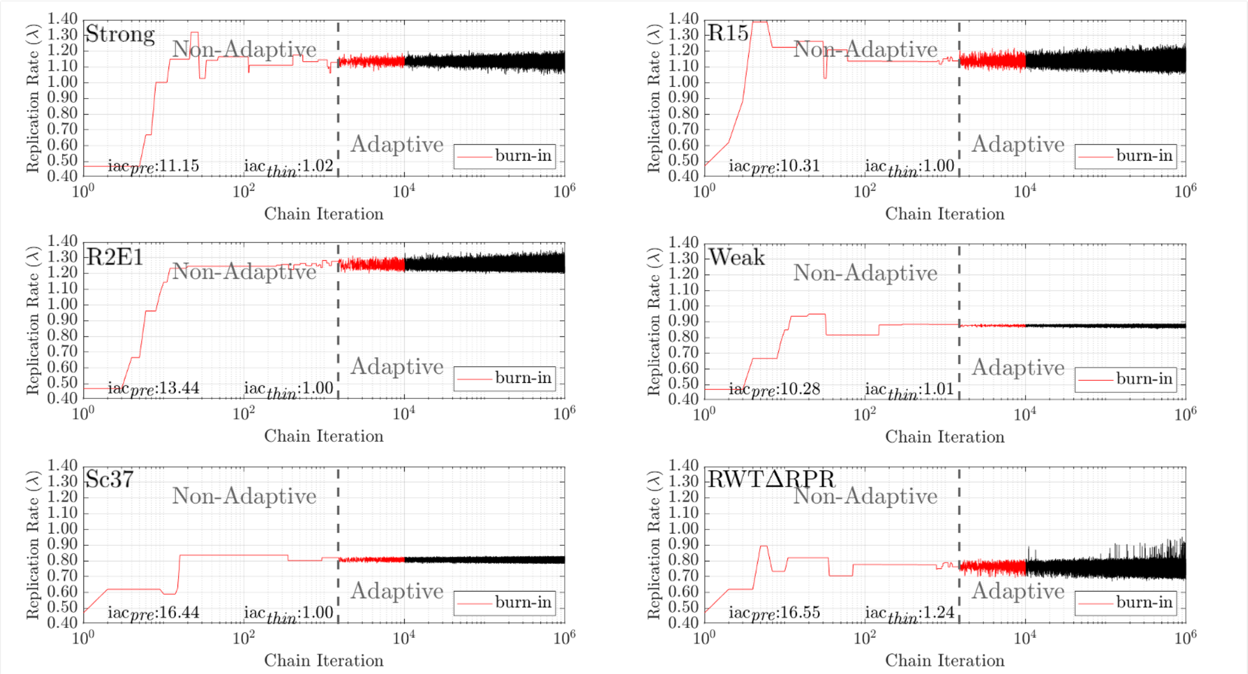

Fig A. Adaptive Metropolis Chain Iterations: Replication Rate. The non-adaptive and adaptive steps are displayed and the burn-in period is highlighted in red. The iac for each chain is presented for both before thinning and dropping the burn-in period (iac$_{pre}$) and after dropping the burn-in period and thinning (iac$_{thin}$).

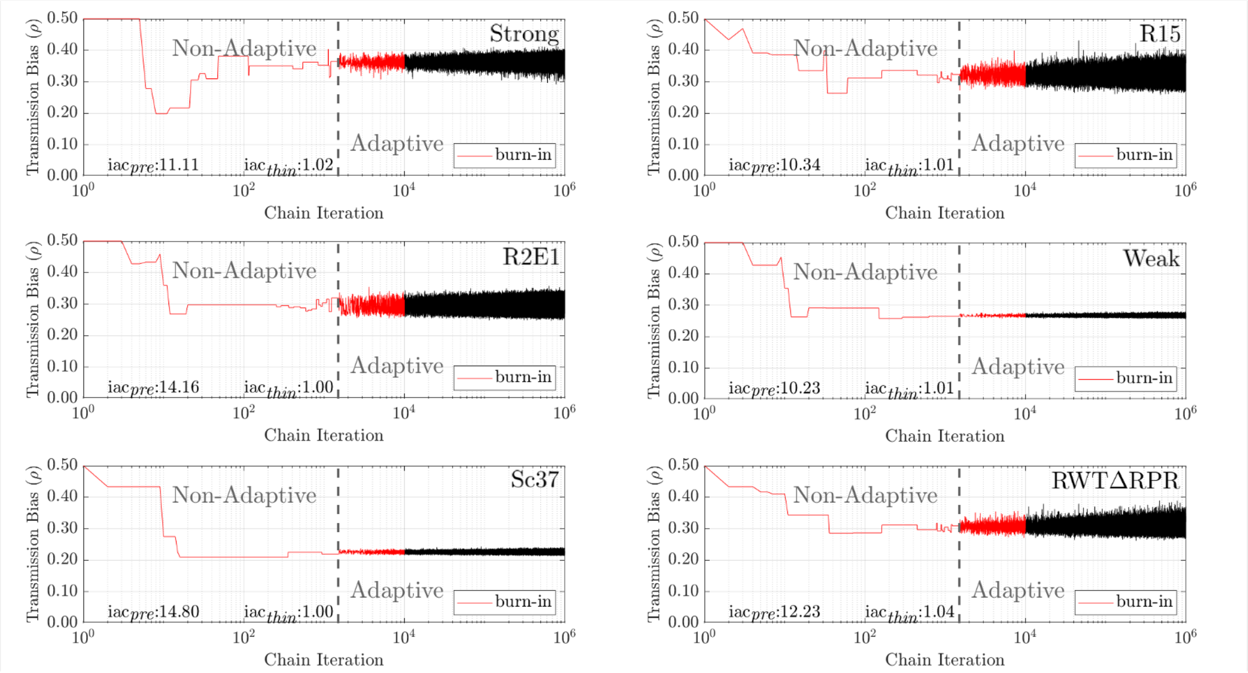

Fig B. Adaptive Metropolis Chain Iterations: Transmission Bias. The non-adaptive and adaptive steps are displayed and the burn-in period is highlighted in red. The iac for each chain is presented for both before thinning and dropping the burn-in period (iac$_{pre}$) and after dropping the burn-in period and thinning (iac$_{thin}$).

Tables of parameter estimates

Prion Variant	T_0		T_1		T_2		T_3		T_4	
	Z_S	Z_A	Z_S	Z_A	Z_S	Z_A	Z_S	Z_A	Z_S	Z_A
Weak	0.71	0.88	0.56	0.62	0.68	0.81	0.58	0.72	0.44	0.51
Sc37	0.48	0.51	0.55	0.59	0.62	0.63	0.81	0.91	0.81	1.11
RWTΔRPR	0.77	0.80	0.96	0.99	1.08	1.43	0.58	0.62	0.96	0.92
Strong	0.78	0.86	1.09	1.17	1.19	1.33	0.93	1.06	0.85	1.06
R15	0.79	0.84	1.13	1.20	1.17	1.39	0.99	1.12	0.83	0.85
R2E1	1.23	1.26	1.79	2.01	0.92	1.17	0.38	0.53	0.62	0.54

Table A. Replication Rate (λ) by T_Δ: Raw Data.

Prion Variant	T_0		T_1		T_2		T_3		T_4	
	Z_S	Z_A	Z_S	Z_A	Z_S	Z_A	Z_S	Z_A	Z_S	Z_A
Weak	0.50	0.27	0.50	0.33	0.50	0.32	0.50	0.27	0.50	0.29
Sc37	0.50	0.28	0.50	0.29	0.50	0.26	0.50	0.25	0.50	0.25
RWTΔRPR	0.50	0.36	0.50	0.37	0.50	0.23	0.50	0.28	0.50	0.30
Strong	0.50	0.31	0.50	0.35	0.50	0.32	0.50	0.32	0.50	0.33
R15	0.50	0.30	0.50	0.31	0.50	0.29	0.50	0.33	0.50	0.31
R2E1	0.50	0.27	0.50	0.19	0.50	0.29	0.50	0.33	0.50	0.37

Table B. Division Bias (ρ) by T_Δ: Raw Data.

Prion Variant	T_0		T_1		T_2		T_3		T_4	
	Z_S	Z_A	Z_S	Z_A	Z_S	Z_A	Z_S	Z_A	Z_S	Z_A
Weak	0.71	0.89	0.56	0.62	0.68	0.81	0.58	0.72	0.45	0.51
Sc37	0.65	0.81	0.46	0.55	0.51	0.59	0.67	0.90	0.51	0.63
RWTΔRPR	0.82	0.82	0.89	0.87	0.91	0.86	0.80	0.77	1.06	1.07
Strong	0.78	0.86	1.08	1.13	1.29	1.43	0.73	0.80	0.94	1.12
R15	0.81	0.84	1.09	1.14	1.10	1.19	1.04	1.18	0.83	0.85
R2E1	1.21	1.25	1.73	2.02	0.79	0.87	0.52	0.66	0.71	0.77

Table C. Replication Rate (λ) by T_Δ: Data Filtered for Outliers.

Prion Variant	T_0		T_1		T_2		T_3		T_4	
	Z_S	Z_A	Z_S	Z_A	Z_S	Z_A	Z_S	Z_A	Z_S	Z_A
Weak	0.50	0.28	0.50	0.33	0.50	0.32	0.50	0.27	0.50	0.29
Sc37	0.50	0.23	0.50	0.30	0.50	0.26	0.50	0.26	0.50	0.33
RWTΔRPR	0.50	0.43	0.50	0.40	0.50	0.32	0.50	0.31	0.50	0.32
Strong	0.50	0.32	0.50	0.36	0.50	0.30	0.50	0.33	0.50	0.31
R15	0.50	0.31	0.50	0.32	0.50	0.31	0.50	0.32	0.50	0.31
R2E1	0.50	0.29	0.50	0.19	0.50	0.33	0.50	0.36	0.50	0.42

Table D. Division Bias (ρ) by T_Δ: Data Filtered for Outliers.

Prion Variant	T_0	T_1	T_2	T_3	T_4
Weak	0	1	2	3	4
Sc37	0	1	2	3	4
RWTΔRPR	0	1	2	3	4
Strong	0	0.75	1	1.5	2
R15	0	0.75	1	1.5	2
R2E1	0	0.75	1	1.5	2

Table E. T_Δ key for each of the six variants. $T_0 = 0$ is the start of the experiment for all six variants. All values presented are in units of hours.

$\boldsymbol{\theta}$		8 Samples/Hour		16 Samples/Hour		32 Samples/Hour	
λ	ρ	λ	ρ	λ	ρ	λ	ρ
0.5	0.2	(0.49,0.52)	(0.19,0.21)	(0.49,0.52)	(0.19,0.21)	(0.50,0.51)	(0.19,0.20)
	0.3	(0.49,0.52)	(0.29,0.32)	(0.49,0.51)	(0.29,0.31)	(0.49,0.51)	(0.30,0.31)
	0.4	(0.49,0.52)	(0.38,0.42)	(0.49,0.51)	(0.39,0.41)	(0.50,0.51)	(0.39,0.41)
	0.5	(0.49,0.50)	(0.48,0.49)	(0.50,0.50)	(0.49,0.50)	(0.50,0.50)	(0.49,0.50)
0.7	0.2	(0.69,0.72)	(0.19,0.21)	(0.69,0.72)	(0.19,0.21)	(0.70,0.71)	(0.19,0.20)
	0.3	(0.69,0.72)	(0.28,0.32)	(0.69,0.71)	(0.29,0.31)	(0.69,0.70)	(0.30,0.31)
	0.4	(0.69,0.71)	(0.38,0.42)	(0.69,0.71)	(0.39,0.41)	(0.70,0.71)	(0.39,0.41)
	0.5	(0.69,0.70)	(0.48,0.49)	(0.70,0.70)	(0.48,0.50)	(0.70,0.70)	(0.49,0.50)
0.9	0.2	(0.89,0.92)	(0.19,0.21)	(0.89,0.92)	(0.19,0.21)	(0.90,0.91)	(0.19,0.20)
	0.3	(0.88,0.92)	(0.29,0.32)	(0.89,0.91)	(0.29,0.31)	(0.89,0.90)	(0.30,0.31)
	0.4	(0.89,0.91)	(0.39,0.42)	(0.89,0.91)	(0.39,0.41)	(0.90,0.91)	(0.39,0.41)
	0.5	(0.89,0.90)	(0.48,0.49)	(0.90,0.90)	(0.49,0.50)	(0.90,0.90)	(0.49,0.50)

Table F. Credible Intervals (95%) for Parameter Estimates of Simulated Data: Filtered for Outliers. The table summarizes the parameter inference results for twelve (λ, ρ) parameter pairs and three sampling rates using data simulated from the ATP model (see methods section for details).

Model weights

Tables G and H present the model weights for every model and dataset combination using the formulation presented in the methods section of the main text. Note that the model weights for each row and across both tables sum to 100%.

Prion Variant	T_0		T_1		T_2		T_3		T_4	
	Z_S	Z_A	Z_S	Z_A	Z_S	Z_A	Z_S	Z_A	Z_S	Z_A
Weak (1/2)	0.00	**99.34**	0.00	0.00	0.00	0.00	0.00	0.00	0.00	0.00
Sc37 (1/2)	0.00	0.00	0.00	0.00	0.00	0.00	0.00	0.00	0.00	0.00
RWTΔRPR (1/2)	0.00	0.00	0.00	0.00	0.00	0.00	0.00	0.00	0.00	0.00
Strong (1/2)	0.00	0.00	0.00	0.00	0.00	0.00	0.00	0.00	0.00	0.00
R15 (1/2)	0.00	0.00	0.00	0.00	0.00	0.00	0.00	0.00	0.00	0.00
R2E1 (1/2)	0.00	0.00	0.00	0.00	0.00	0.00	0.00	0.00	0.00	0.00

Table G. Percent Model Weights by T_Δ: Raw Data.

Prion Variant	T_0		T_1		T_2		T_3		T_4	
	Z_S	Z_A	Z_S	Z_A	Z_S	Z_A	Z_S	Z_A	Z_S	Z_A
Weak (2/2)	0.00	**0.66**	0.00	0.00	0.00	0.00	0.00	0.00	0.00	0.00
Sc37 (2/2)	0.00	**100**	0.00	0.00	0.00	0.00	0.00	0.00	0.00	0.00
RWTΔRPR (2/2)	0.00	0.00	0.00	0.00	0.00	0.00	0.00	**100**	0.00	0.00
Strong (2/2)	0.00	0.00	0.00	**100**	0.00	0.00	0.00	0.00	0.00	0.00
R15 (2/2)	0.00	0.00	0.00	**100**	0.00	0.00	0.00	0.00	0.00	0.00
R2E1 (2/2)	0.00	**100**	0.00	0.00	0.00	0.00	0.00	0.00	0.00	0.00

Table H. Percent Model Weights by T_Δ:Data Filtered for Outliers.

Experimental Data

The following tables, Tables I-N, present the experimental propagon counts for six prion variants obtained experimentally from propagon recovery experiments as discussed in the main text.

Time (hrs)	Propagon Counts
0	36, 26, 22, 18, 14, 6, 14, 34, 15, 2, 8, 4, 10, 7
1	48, 33, 32, 41, 48, 33, 12, 14, 30, 45, 58, 60, 52, 34, 85<br>6, 8, 12, 9
2	72, 46, 64, 60, 52, 20, 12, 47, 24, 33, 64, 68, 36, 48, 78<br>34, 33, 23, 12, 5
3	103, 59, 76, 135, 60, 94, 32, 121, 63, 186, 57, 59, 47, 72, 108<br>15, 10, 8, 11, 26
4	93, 221, 100, 107, 62, 146, 213, 91, 188, 138, 230, 52, 17, 104, 28<br>27, 36
5	180, 163, 208, 65, 136, 194, 23, 59, 58, 83, 199, 60, 291, 36, 41<br>76, 48, 50
6	238, 170, 160, 122, 80, 19, 58, 162, 42, 285, 147, 186, 321
7	262, 279, 236, 221, 129, 81, 103, 182, 152, 52
8	169, 179, 256, 134, 131, 163, 157, 132, 178, 169

Table I. Aggregate Counts for Weak Variant.

Time (hrs)	Propagon Counts
0	100, 41, 99, 12, 26, 4, 27, 39, 5, 59, 19, 20, 25, 16, 5<br>9, 1, 3, 7, 23, 10, 4, 3, 13
1	28, 7, 57, 15, 53, 15, 27, 49, 43, 49, 53, 68, 32, 4, 2<br>2, 1, 4, 2
2	65, 80, 52, 50, 21, 18, 9, 21, 11, 21, 43, 40, 37, 79, 1<br>32, 17, 1, 10, 13
3	49, 70, 29, 28, 25, 30, 20, 37, 23, 25, 51, 24, 62, 45, 109<br>46, 44, 48, 7, 12, 9, 2, 18, 19, 15
4	97, 48, 85, 58, 18, 46, 73, 75, 103, 145, 59, 8, 4, 23, 11<br>2, 4, 1, 159
5	33, 108, 101, 36, 48, 35, 11, 87, 33, 44, 33, 87, 53, 287, 261<br>322, 106, 267, 24, 15, 8, 17, 7, 38
6	139, 49, 33, 43, 77, 58, 317, 197, 107, 130
7	46, 395, 185, 240, 46, 38, 343, 210, 181, 106, 167, 132
8	314, 345, 251, 141, 53, 40, 126, 95, 196, 98, 82, 263, 199

Table J. Aggregate Counts for Sc37 Variant.

Time (hrs)	Propagon Counts
0	3, 3, 1, 2, 2, 2, 6, 3, 7, 3, 3
1	1, 5, 5, 2, 3, 1, 4, 2, 5
2	1, 3, 8, 2, 6, 7, 3, 8, 4
3	4, 63, 3, 9, 12, 4, 13, 7, 9, 14, 12
4	7, 8, 39, 2, 17, 5, 17, 6, 18, 17, 17
5	36, 48, 19, 16, 33, 49, 18, 23, 11, 28, 12
6	25, 5, 43, 41, 30, 16, 48, 58, 47, 92, 72
7	63, 63, 111, 65, 65, 84

Table K. Aggregate Counts for RWTΔRPR Variant.

Time (hrs)	Propagon Counts
0	2, 8, 6, 7, 11, 8, 1, 5, 2, 2, 11, 9, 14, 7, 6<br>8, 8, 3, 7, 4, 7, 5, 17, 5, 2, 1, 4
0.75	5, 3, 6, 3, 9
1	8, 13, 5, 4, 12, 2, 3, 3, 7, 4, 11, 6, 8, 12, 2<br>2, 5, 3, 3, 4
1.5	6, 1, 1, 8, 4, 7
2	15, 14, 2, 4, 6, 9, 10, 5, 6, 10, 14, 11, 24, 39, 3<br>7, 9, 2, 18, 15, 32
2.25	16, 19, 15, 9
3	30, 24, 14, 15, 23, 26, 19, 34, 30, 74, 62, 9, 20, 28, 45<br>57, 63, 25, 34, 60, 19, 80, 37, 20, 74, 32, 74
3.75	54, 61, 83, 37, 157, 88
4	29, 31, 60, 26, 51, 54, 72, 66, 14, 74, 82, 132, 149, 66, 194<br>54, 41, 73, 61, 81, 79
5	60, 80, 116, 54, 24, 138, 109, 24, 79, 192, 387, 276, 134, 217, 128<br>96, 155, 148, 123, 213
6	77, 93, 91, 98, 80, 76, 119, 234, 190, 145, 182
7	155, 179, 188, 89, 166

Table L. Aggregate Counts for Strong Variant.

Time (hrs)	Propagon Counts
0	3, 2, 2, 4, 7, 3, 2, 1, 4, 2, 9, 1, 5, 4, 13<br>7, 4, 14, 4, 1, 2, 1, 11
0.75	3, 1, 11, 1
1	2, 4, 7, 2, 4, 5, 5, 8, 3, 4, 2, 3, 8, 5, 2<br>2, 6, 5, 3, 2
1.5	1, 3, 4, 3, 4
2	3, 4, 9, 4, 3, 1, 3, 17, 4, 13, 22, 24, 10, 21, 15<br>13, 16, 7, 4, 8, 2, 5, 8
2.25	7, 9, 23, 8, 6, 5
3	7, 4, 36, 23, 6, 8, 31, 9, 61, 3, 5, 23, 11, 24, 26<br>10, 9, 24, 100, 34, 28, 37, 43, 17, 46, 39, 14, 29
3.75	30, 19, 45, 27, 65
4	52, 56, 13, 65, 77, 105, 45, 32, 11, 49, 92, 8, 130, 51, 103<br>34, 42, 45, 37
5	58, 88, 129, 75, 71, 31, 95, 15, 165, 149, 79, 117, 110, 201, 57<br>103, 154
6	84, 101, 65, 87, 137, 60, 52, 146, 147
7	188, 166, 75, 150

Table M. Aggregate Counts for R15 Variant.

Time (hrs)	Propagon Counts
0	9, 6, 2, 10, 3, 1, 5, 6, 8, 4, 11, 19, 2, 13, 13<br>10, 7, 2, 2, 9, 4, 3
0.75	3, 9, 6, 2, 5
1	7, 21, 2, 11, 9, 4, 6, 17, 13, 7, 1, 3, 2, 13
1.5	14, 5, 2, 31, 4
2	32, 23, 71, 20, 93, 42, 119, 112, 89, 15, 55, 92, 156, 134, 35<br>17
2.25	14, 33, 21, 67
3	157, 191, 156, 111, 207, 193, 116, 131, 85, 134, 361, 420, 368, 413, 274<br>197, 149, 86, 106, 167, 119, 134, 216
3.75	298, 175, 194, 249, 213, 101
4	200, 77, 134, 136, 140, 256, 389, 254, 501, 194, 315, 107, 169, 120
5	122, 158, 33, 189, 222, 205, 438, 379, 619, 388, 402, 155, 127, 112, 190<br>204, 323, 186, 185, 181
6	35, 166, 225, 228, 198, 155

Table N. Aggregate Counts for R2E1 Variant.
